# Characterization of Different Substituted Carboxymethyl Starch Microgels and Their Interactions with Lysozyme

**DOI:** 10.1371/journal.pone.0114634

**Published:** 2014-12-09

**Authors:** Bao Zhang, Han Tao, Benxi Wei, Zhengyu Jin, Xueming Xu, Yaoqi Tian

**Affiliations:** 1 The State Key Laboratory of Food Science and Technology, Jiangnan University, Wuxi, China; 2 School of Food Science and Technology, Jiangnan University, Wuxi, China; 3 Synergetic Innovation Center of Food Safety and Nutrition, Jiangnan University, Wuxi, China; Kermanshah University of Medical Sciences, Islamic Republic of Iran

## Abstract

A carboxymethyl starch (CMS) microgel system was prepared for the control of uptaking and releasing proteins (lysozyme). The physicochemical properties of microgels in various degrees of substitution (DS) were determined by thermal gravimetric analysis (TGA), swelling degree, and rheological analysis. The microgel particle size mostly ranged from 25 µm to 45 µm. The result obtained from the TGA studies indicated that carboxymethylation decreased the thermal stability of starch, but crosslinking increased the thermal stability of CMS. The CMS microgels showed typical pH sensitivity, and the swelling degree of microgel increased with the increasing of DS and pH, because of the large amounts of carboxyl group ionization. The samples (2.25%) could behave as viscoelastic solids since the storage modulus was larger than the loss modulus over the entire frequency range. The protein uptake increased with increasing pH and DS at low salt concentration. The optimal pH shifted to lower pH with increasing ionic strength. The saturated protein uptake decreased with increasing ionic strength at each pH. The protein was easily released from the microgel with high pH and high salt concentration.

## Introduction

Hydrogels can often be classified either macrogels or microgels depending on their sizes. The former is also termed bulk gels, with size ranging from millimeters to a few centimeters. Microgels mostly have average diameters between 50 nm and 100 µm and are stabilized in continuous medium to form stable dispersions [1], [2]. Microgels and the corresponding macrogels share the same chemical compositions and thermodynamic properties, but they exhibit many distinct differences. For example, microgel particles have low viscosity dispersions, whereas macrogels are viscoelastic solids. Microgels also rapidly respond to environmentally triggered changes in swelling and molecule binding that enable their use in controlled and regulated applications [3], [4]. Microgels are valued for their functionality and ability to tune physical properties in industrial applications, including oil recovery, paint and surface coating, controlled drug delivery, cosmetics, personal care, pharmaceuticals, and foods [5].

Controlled uptake and release of functional compounds, such as peptides and proteins from microgel carriers, have been extensively studied [6]–[9]. The properties of these compounds enable them to respond to external stimuli, such as temperature, pH, ionic strength, solvent, applied electric or magnetic fields. Microgels from natural polymers are particularly interesting because of their biodegradability and biocompatibility. Microgels from high amylose starch have been synthesized and investigated for controlled drug release [10]–[12]. However, a disadvantage of these microgels is that it is difficult to control the charge density on the polysaccharides and therefore the electrostatic binding of proteins and peptides. Charged microgels typically swell extensively in water because of the high osmotic swelling pressure from counterions inside the network, and the fixed charges give them the capacity to bind large amounts of opposite charged proteins [13], [14]. Recently, microgels based on phosphate cross-linked oxidized potato starch polymer, which completely control the polysaccharide charge density, have been extensively investigated [15]–[17]. Similar to oxidized starch, carboxymethyl starch (CMS) also has a negative charge. The polysaccharides crosslinked with associated microspheres from carboxymethyl pullulan have been synthesized, and their interactions with lysozyme have been investigated [18].

Sodium trimetaphosphate (STMP), a low toxicity salt with no adverse effects on humans, is an effective cross-linking agent for starches [19], [20]. The cross-linking reaction occurs through the hydroxyl groups of the polysaccharide and leads to ester linkages. This interesting coupling agent was chosen in the preparation of CMS microgel because of its non-reactive property with carboxylic groups, which are free for further modifications.

This study focuses on the synthesis and characterization of different substituted-CMS microgels. The physicochemical properties of these microgels in various degrees of substitution (DS) were determined by thermogravimetric analysis (TGA), swelling degree, and rheological analysis. The swelling degree (SD) of the microgels was examined in solutions with different pH values at an ionic strength of 0.05 M. Lysozyme uptaking and releasing properties from the microgels were also investigated.

## Materials and Methods

### Materials

Waxy corn starch (amylose content 0.5%, M_w_ = 2.31×10^8^ Da) was kindly donated by Tianjin Tingfung Starch Development Co., Ltd. (Tianjin, China). Carboxymethyl starch (CMS) was prepared using waxy corn starch according to the reported method in our laboratory [21]. The degree of substitution (DS) of CMS samples was determined via determination of the carboxyl content according to Liu et al. [22]. The DS of CMS were 0.31, 0.67, and 0.95, and the moisture contents of CMS were 10.3%, 11.2%, and 10.6%, respectively. Sodium trimetaphosphate (STMP) was purchased from Sigma Aldrich Trading Co. Ltd. (Shanghai, China) and of analytical grade. All other chemicals and reagents were purchased from Sinopharm Chemical Reagent Co., Ltd. (Suzhou, China) and of analytical grade.

### Microgel preparation

**Figure 1:**
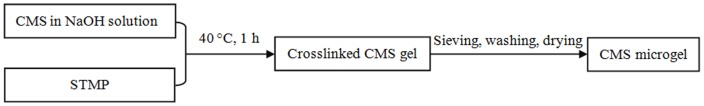
Scheme of preparing CMS microgels.

Microgels were prepared by cross-linking the different substituted CMS polymers with STMP according to previous methods [16]. The reaction scheme for crosslinking CMS microgels was depicted in Fig. 1. CMS polymer (10 g) was dissolved in 45 mL sodium hydroxide solution for 30 min. The cross-linker (STMP) was then added to the polymer solution. The mixture was heated at 40°C for 1 h without stirring, resulting in a gel formation. The weight ratio of STMP to sodium hydroxide was 3∶1, and the weight ratio of STMP to CMS was 0.2. The gel was then stored overnight in a cold room at 4°C. The whole piece of gel was ground through a sieve (1 mm) covered with a nylon cloth of 200 mesh to obtain reasonably uniform microgel particles. The gels were washed three times with dionized water and sieved again using the aforementioned nylon cover to remove salts. The microgel particles were washed three times in 100% ethanol to remove water and three times in 100% acetone to remove ethanol and last traces of water. The microgel particles were then dried in an oven at 40°C overnight and ground to achieve small and homogenous particles using a sieve of 200 mesh.

### Size distribution of microgel particles

The size distribution of the different substituted microgel particles in a suspension was determined using a Malvern MasterSizer 2000 (Malvern Instrument, Ltd., UK). The sample concentration was within the range of the instrument specifications. Prior to measurement, suspensions were sonicated for 15 min to attain finely dispersed gel particles.

### Thermogravimetry analysis (TGA)

TGA was performed on a thermogravimetric analyzer (TGA-SDTA851e; Mettle Toledo Co. Ltd., Switzerland). Samples of about 5 mg were heated from 25°C to 500°C at a heating rate of 10°C/min under N_2_ at 20 mL/min during the analysis.

### Swelling degree (SD) measurements

The dried gel powder (50 mg, dry basis) was mixed with deionized water/buffer (25 mL) in 50-mL centrifuge tubes at room temperature. The swollen microgel was immersed in buffer for 24 h to obtain equilibrium, and the tubes were then centrifuged at 2,000×g for 30 min. The precipitates were immediately weighed, and SD was estimated as follows:
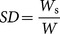
where W_s_ is the weight of the sediment and W is the weight of the dry gel (db, mg).

### Determination of rheological properties

The rheological properties of all samples were determined using a stress-controlled rheometer (AR 2000; TA Instruments Inc., New Castle, DE, USA) with an aluminum parallel plate geometry (50 mm diameter, 1.00 mm gap). The samples were carefully weighed (0.225 g) and mixed with 9.775 mL of deionized water. Small amplitude oscillatory tests were performed at a frequency range of 0.1 Hz to 100 Hz. The selected strain amplitude for the frequency sweep measurements was 0.1%, which was in the linear viscoelastic region for all samples. Thus, the dynamic viscoelastic functions, such as the dynamic shear storage modulus (G′) and loss modulus (G″), were measured as a function of frequency.

### Saturated protein uptake capacity

Dry gel particles (3 mg) were suspended in 7 mL of buffer at various with 3 mL of 10 mg/mL protein solution and gently stirred for 4 h. Subsequently, the samples were centrifuged at 12000 rpm for 10 min, and lysozyme concentration in the supernatant was measured by the absorbance at wavelength 280 nm using a UV spectrophotometer (TU-1900, Rayleigh Analytical Instruments, Beijing, China). The total protein adsorption at saturation level Γ_sat_ (mg protein/mg dry gel) in the microgel particles was calculated from the mass balance.
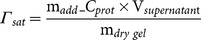
where V_supernatant_ is the volume of the supernatant after centrifugation, C_prot_ is the lysozyme concentration in the supernatant, m_add_ is the total lysozyme added, and m_dry gel_ is the weight of the added dry microgel.

### Protein release under dilution

Samples of microgel-protein mixtures of different pH values were prepared in the same approach as previously described, stirred for 4 h, and centrifuged at 12000 rpm for 10 min. The sediments (protein–gel complexes) were diluted with 10 mL of fresh buffer and mildly stirred for another 4 h. After the second centrifugation, the absorbance in the supernatants was determined. The percentage of protein released P_rel_ was calculated from the measured protein concentration C_prot_ as follows: 
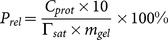



### Statistical analysis

Analyses of the samples were completed in triplicate and standard deviations were reported. The means were compared with Tukey's test (to a 5% level of significance) using analysis of variance (ANOVA).

## Results and Discussion

### Size distribution of microgels

**Figure 2:**
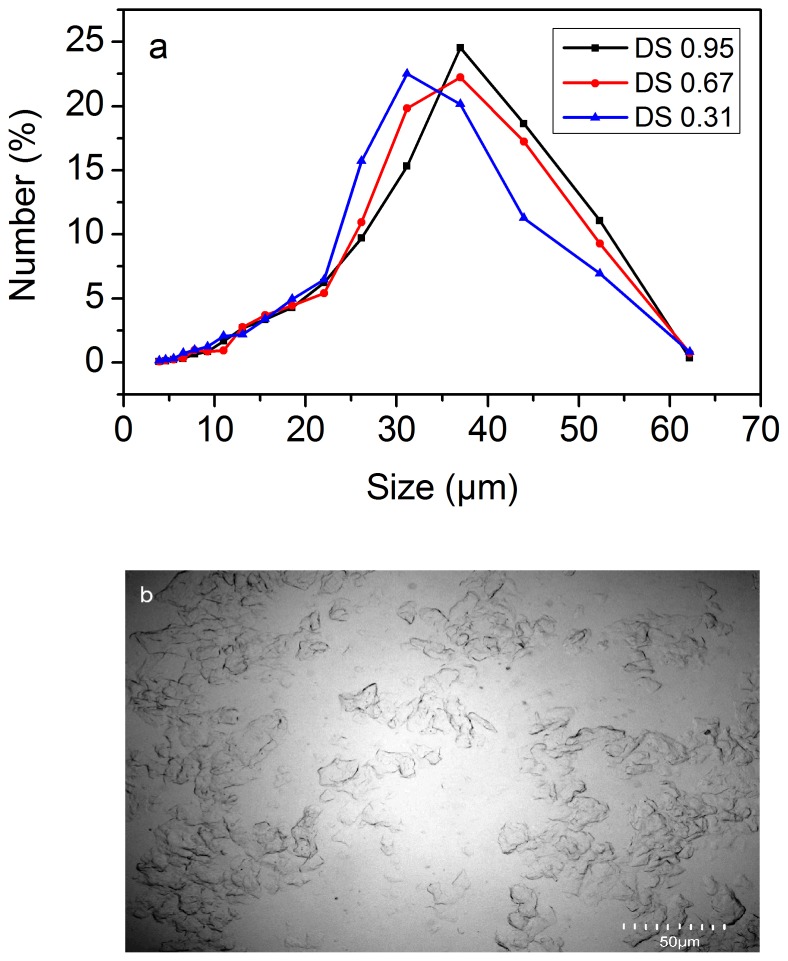
(a) Numbers of starch polymer microgel particles in water vs. their size (volume weighted mean diameter in µm), for various degrees of substitution (DS); (b) Optical microscopic image of microgels with DS  = 0.31 dispersed in water. Scale bar shown in graph is 50 µm.

Fig. 2a shows the distributions of the particle size of microgels for various DS. The particle size of microgels ranged from 2 µm to 65 µm and mostly concentrated in the range of 25 µm to 45 µm. The curves of the particle size distributions became larger with the increase in DS because higher DS have larger amounts of carboxyl groups that resulted in stronger electrostatic repulsion. Furthermore, Fig. 2b shows an optical microscopic picture ofmicrogels with DS = 0.31. Obviously, the shapes of microgels were irregular.

### TGA

**Figure 3:**
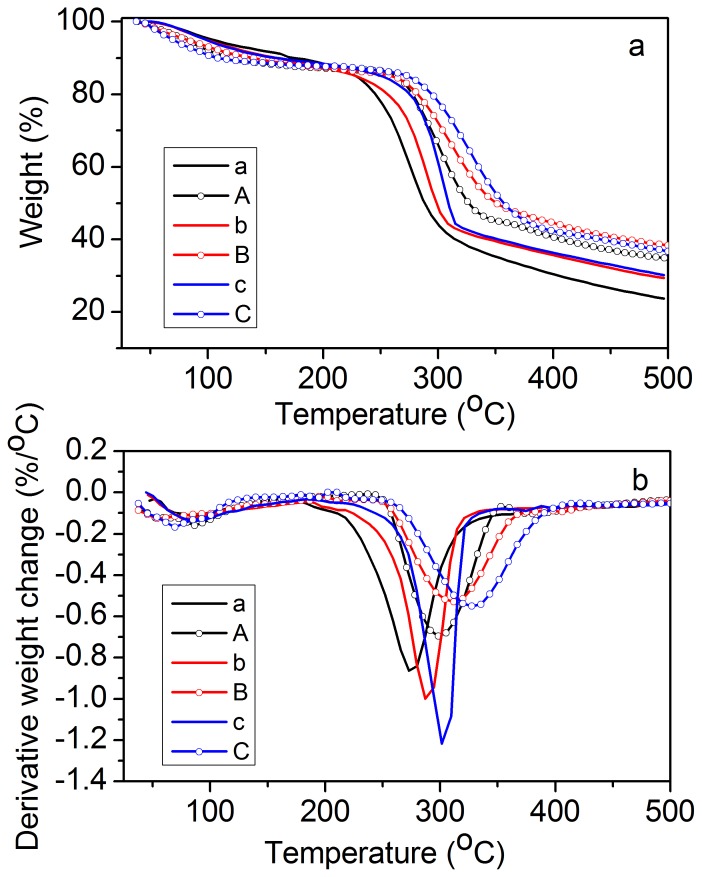
TGA and the derivative TGA curves of CMS and microgels for various degrees of substitution (DS). a, b, and c are CMS of DS 0.31, 0.67, and 0.95, respectively. A, B, and C are microgels of DS 0.31, 0.67, and 0.95, respectively.

The thermal properties of the tested compounds were investigated at a temperature range of 25°C to 500°C to evaluate the water content of the samples and to verify the degradation process in CMS and corresponding microgels with various DS. The TGA and DTG curves for CMS and the corresponding microgels for various DS were presented in Fig. 3. The decrease in weight from 50°C to 120°C was related to the loss of water, and the second decrease corresponded to its decomposition [23]. Water was the main product of decomposition at temperatures below 300°C. Further heating up to 500°C resulted in carbonization and ash formation. A slight decrease was observed in the initial thermal stability of CMS with increasing DS. The DTG curve for CMS showed two peaks; the first peak corresponded to the loss of water at around 50°C to 120°C and the second peak corresponded to stage decomposition. A shift of maximum peak toward low temperatures was observed with increasing DS. It had been found that the carboxymethylation decreased the thermal stability of starch materials. This decrease in thermal stability with increasing DS might be attributed to the nature of carboxymethyl. Carboxymethyl had a hydrophilic character that led to decreasing thermal stability of starch macromolecules [24]. Similarly, their corresponding microgels showed the same effect. Furthermore, the thermal stability of microgel was higher than that of corresponding CMS. Considering that the main decomposition mechanism was through the dehydration reaction between the hydroxyl groups, we concluded that a low OH group content on the starch derivative would result in a stable microgel [25]. Some differences in the mass residues of samples after thermal analyses were due to the presence of sodium salt of carboxymethyl groups.

### Swelling capacity

**Figure 4:**
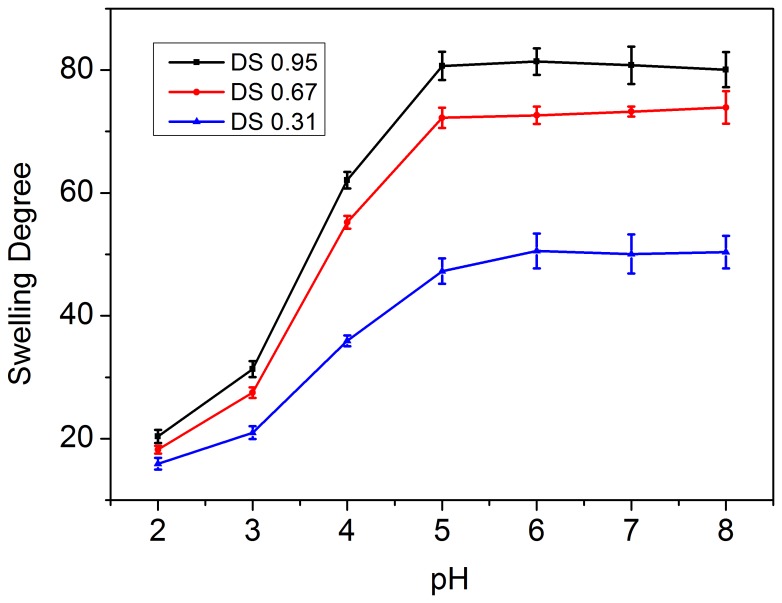
Swelling degree as a function of pH for various degrees of substitution (DS). Buffer conditions: 0.02 M citric acid-phosphate buffer (pH 2–8) and ionic strength of 0.05 M. (Data shown in this figure were expressed as means ± standard deviations of triplicate.)

Fig. 4 shows swelling degree (SD) as a function of pH at an ionic strength 0.05 M for various DS. The percentage of maximum swelling ratio increased from pH 2 to pH 5 and maintained almost constant from pH 5 to pH 8. This result indicated that the synthesized microgels had a pH-sensitive swelling behavior. At pH less than 5, the dissociation of carboxyl groups caused an influx of counterions to balance the charge, resulting in the concentration of ions in the microgels higher than in the surrounding. Therefore, a difference in osmotic pressure and a solution flux into the microgels occurred and led to swelling. The elastic restoring forces resulted in an equilibrium ionic microgel. At pH greater than 5, the dissociation of carboxylic groups was complete, and the repulsion between the polyelectrolyte chains was maximal. The SD of microgels increased with increasing DS at each pH because high DS had large numbers of carboxyl groups that resulted in a strong electrostatic repulsion. The maximum swelling ratios at pH 5 for microgels of DS 0.31, DS 0.67, and DS 0.95 were 47.3, 72.2, and 80.6, respectively. The swelling degrees between these microgels were significant difference at this pH.

### Rheological measurements

**Figure 5:**
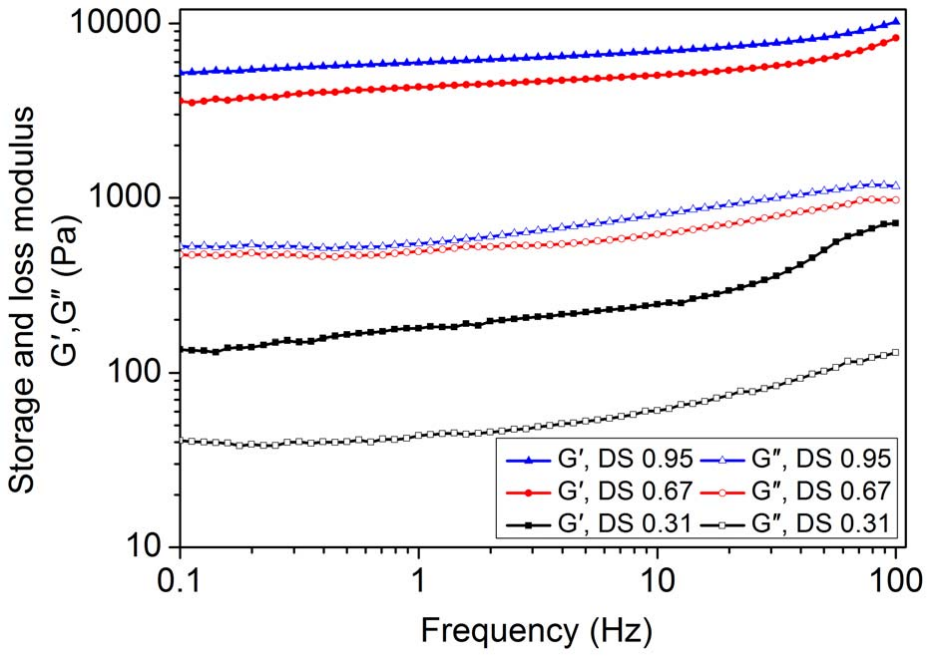
Frequency dependence of G′ and G″ at a constant strain (0.1%) for microgels of various degrees of substitution (DS) for 2.25% (W/W).

Frequency sweep tests were widely used to obtain information about the stability of three-dimensional cross-linked networks [26], [27]. The frequency dependence of the viscoelastic properties of microgels in various DS was shown in Fig. 5. For each sample, G′ and G″ were measured as a function of the frequency from 0.1 Hz to 100 Hz. At a lower frequency, both G′ and G″ were virtually frequency independent but gradually increased with increasing frequency at a higher frequency. Entangled chain of polymer had sufficient time to relax at a low frequency; therefore, the storage modulus maintained almost constant. However, at a high frequency, the chain of microgels failed to rearrange themselves in the time scale of the imposed motion; this rearrangement failure caused the microgels to stiffen and assume a more “solid-like” behavior characterized by an increase in G′ [28]. The gels exhibited typical viscoelastic properties of starch gels. In all cases, the values of storage modulus (G′) were higher than those of loss modulus (G″), and the values of phase shift (tan δ  =  G″/G′) were lower than 1. These results indicated that elastic prevailed over viscous properties, and the swollen sample exhibited mechanical rigidity.

The rheological behavior was different from the microgels of various DS at the same concentration and temperature. The change in viscoelastic properties caused by etherification significantly depended on the amount of carboxyl groups. Microgels with higher DS displayed higher values of storage and loss moduli than those with lower DS. This finding was due to the fact that carboxyl groups were more polar than hydroxyl groups, thus forming stronger microgel bond with water and leading to an increase of the elastic modulus. Microgels with higher DS had higher SD than those with lower DS. Therefore, the increase of G′ might be due to the smaller free water in the sample of higher DS than lower DS, restricting the movement of the chain of microgels and resulting in the increase of G′.

### Saturated protein uptake capacity of the microgels

**Figure 6:**
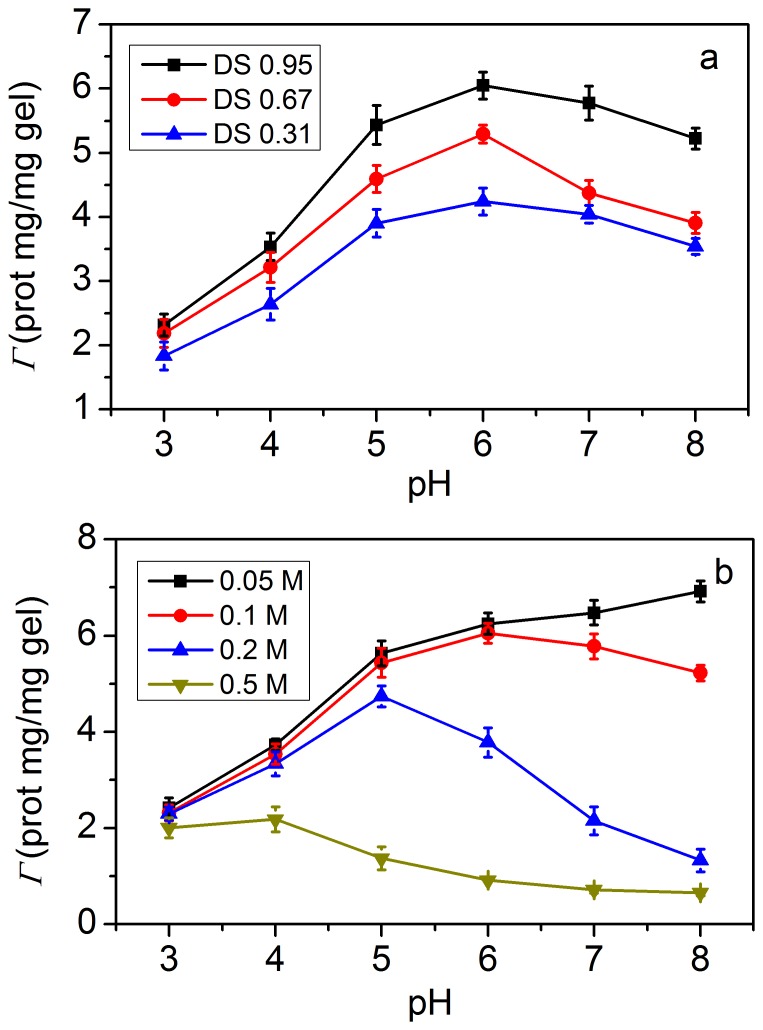
(a) Lysozyme uptake Γ (prot mg/mg gel) as a function of pH citric acid-phosphate buffer at an ionic strength of 0.1 M for microgels of various degrees of substitution (DS); (b) lysozyme uptake Γ (prot mg/mg gel) for the DS 0.95 microgel at various ionic strengths as a function of pH. (Data shown in this figure were expressed as means ± standard deviations of triplicate.)

Fig. 6a shows lysozyme uptake Γ (prot mg/mg gel) as a function of pH citric acid-phosphate buffer at an ionic strength of 0.1 M for microgels of various DS. The pH change provided an opportunity to modify the electrostatic binding between the lysozyme and microgel and controlled the lysozyme loading. The maximal loading capacity of microgels was obtained at pH 6, when both the lysozyme molecule and the carboxylic group of the microgels were completely ionized. Much lower loadings were achieved at pH 3.0 or at pH 8.0, respectively, which was attributed to either partial protonation of the carboxylic groups in the microgels at low pH or deprotonation of positively charged amino group of lysozyme at high pH. The loading capacity of microgel increased with increasing DS. It appeared that the loading capacity was mainly controlled by the number of the carboxylate groups in the microgels available for lysozyme binding.

The ionic strength of the loading medium also affected the uptake capacity of the microgel (Fig. 6b). The uptake capacity of the microgel decreased with increasing ionic strength at each pH. At low ionic strength, charge compensation was a primary force between lysozyme and microgels. However, an increase of the ionic strength of the loading buffer caused by the screening effect decreased the electrostatic interactions between lysozyme and microgels.

### Protein release under dilution

**Figure 7:**
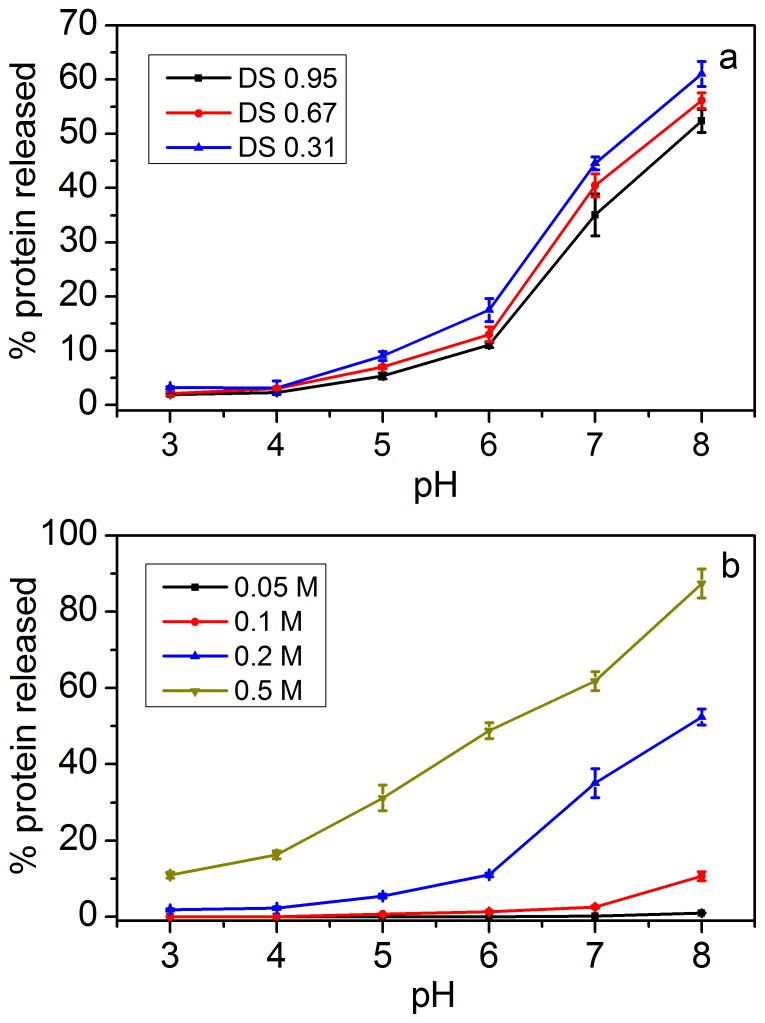
(a) Percentage of lysozyme released from DS 0.31, DS 0.67, and DS 0.95 microgels by dilution with buffer as a function of pH at an ionic strength of 0.2 M; (b) percentage of lysozyme released from DS 0.95 microgel by dilution with buffer as a function of pH at various ionic strengths. (Data shown in this figure were expressed as means ± standard deviations of triplicate.)

Fig. 7a shows the percentage of lysozyme released from DS 0.31, DS 0.67, and DS 0.95 microgels as a function of pH after 4 h equilibrium in buffer solutions at an ionic strength of 0.2 M. The microgels were saturated with lysozyme before the releasing experiment. The degree of protein release from the complexes increased with increasing pH. Non-specificity of differences was demonstrated at pH 3 and pH 4. The positive charge of lysozyme decreased with increasing pH. The binding affinity decreased with increasing pH, resulting in more lysozyme escaping from the microgel. Furthermore, the pore size of microgel increased with increasing pH, and the lysozyme molecules were much easier to release from the microgel. The result also showed that high DS microgel released less lysozyme than low DS microgel. This result was attributed to the high DS microgel, which had more negative charges than low DS, indicating that the binding affinity was higher for high DS microgel than low DS.

The percentage of lysozyme released from DS 0.95 microgel by dilution with buffer as a function of pH at various ionic strengths was presented in Fig. 7b. The percentage of lysozyme release increased with increasing salt concentration, which was due to the screening of electric charges of microgel by the presence of salt. These results revealed that the protein bound with low affinity at high pH and high salt concentration and was easily released by dilution. By contrast, the protein bound strongly that dilution resulted in slight protein release.

## Conclusions

A starch-based microgel incorporating carboxyl was prepared using chemical cross-linking. The microgel particle size mostly ranged from 25 µm to 45 µm. Carboxymethylation decreased the thermal stability of starch, but cross-linking increased the thermal stability of CMS. The SD of microgel was influenced by pH; SD increased as the pH increasing because of loosening of the microgel structure resulting from the repulsion of ionized carboxyl groups. The ability to absorb water increased with the increase of DS of all the CMS microgels. The capability of microgels to store large amounts of oppositely charged molecules in a small volume made them potential drug delivery systems, particularly for macromolecular drugs. Furthermore, promising results were obtained demonstrating the possibility of pH-triggered release of cationic protein and peptide drugs from anionic microgels.
