# Correction: Characterization of Different Substituted Carboxymethyl Starch Microgels and Their Interactions with Lysozyme

**DOI:** 10.1371/journal.pone.0119766

**Published:** 2015-03-17

**Authors:** The PLOS ONE Staff

There is an error in the first sentence of the “Saturated protein uptake capacity” subsection of the Materials and Methods. The correct sentence is: Dry gel particles (3 mg) were suspended in 7 mL of buffer at various pH with 3 mL of 10 mg/mL protein solution and gently stirred for 4 h.

There are errors in the legend for Fig. 3, “TGA and the derivative TGA curves of CMS and microgels for various degrees of substitution (DS).” Fig. 3 and the complete, correct Fig. 3 legend are below.

**Fig 3:**
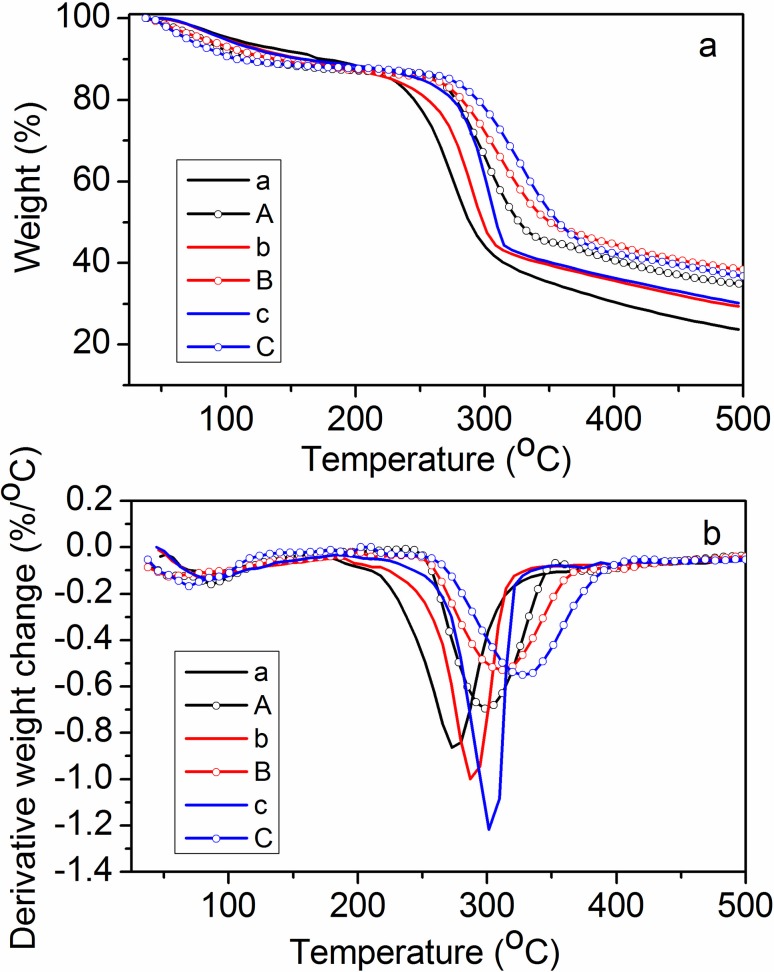
TGA and the derivative TGA curves of CMS and microgels for various degrees of substitution (DS). a, b, and c are CMS of DS 0.95, 0.67, and 0.31, respectively. A, B, and Care microgels of DS 0.95, 0.67, and 0.31, respectively.
